# Supplementary material for: Causal Relationship between Aging and Anorexia Nervosa: A White-Matter-Microstructure-Mediated Mendelian Randomization Analysis
Source: Biomedicines. 2024 Aug 16;12(8):1874. doi: 10.3390/biomedicines12081874 (PMC11351342; doi:10.3390/biomedicines12081874)
Supplement: Supplementary file 1 [file biomedicines-12-01874-s001.zip › Supplementary Figure/Figure S4/Singe SNP plot of MR analysis for TL to ubm-b-1984.pdf]

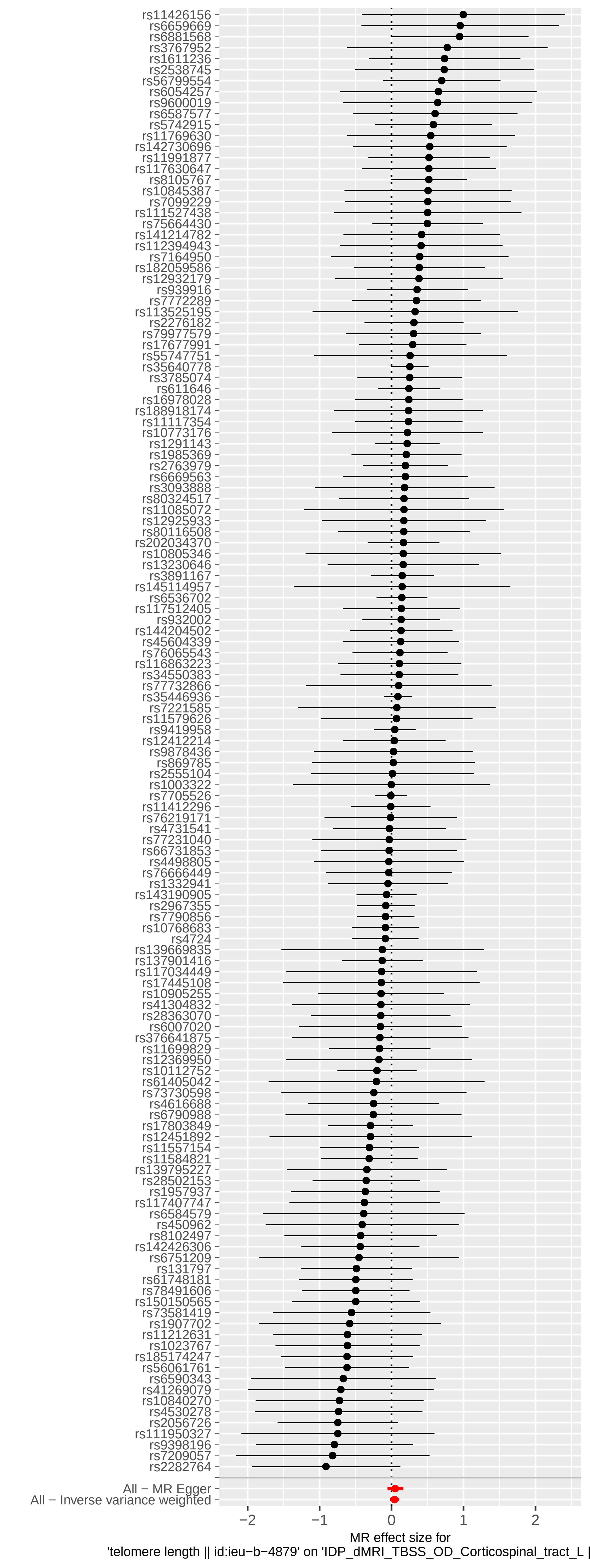

rs11426156
rs6659669
rs6881568
rs3767952
rs1611236
rs2538745
rs56799554
rs6054257
rs9600019
rs6587577
rs5742915
rs11769630
rs142730696
rs11991877
rs117630647
rs8105767
rs10845387
rs7099229
rs111527438
rs75664430
rs141214782
rs112394943
rs7164950
rs182059586
rs12932179
rs939916
rs7772289
rs113525195
rs2276182
rs79977579
rs17677991
rs55747751
rs35640778
rs3785074
rs611646
rs16978028
rs188918174
rs11117354
rs10773176
rs1291143
rs1985369
rs2763979
rs6669563
rs3093888
rs80324517
rs11085072
rs12925933
rs80116508
rs202034370
rs10805346
rs13230646
rs3891167
rs145114957
rs6536702
rs117512405
rs932002
rs144204502
rs45604339
rs76065543
rs116863223
rs34550383
rs77732866
rs35446936
rs7221585
rs11579626
rs9419958
rs12412214
rs9878436
rs869785
rs2555104
rs1003322
rs7705526
rs11412296
rs76219171
rs4731541
rs77231040
rs66731853
rs4498805
rs76666449
rs1332941
rs143190905
rs2967355
rs7790856
rs10768683
rs4724
rs139669835
rs137901416
rs117034449
rs17445108
rs10905255
rs41304832
rs28363070
rs6007020
rs376641875
rs11699829
rs12369950
rs10112752
rs61405042
rs73730598
rs4616688
rs6790988
rs17803849
rs12451892
rs11557154
rs11584821
rs139795227
rs28502153
rs1957937
rs117407747
rs6584579
rs450962
rs8102497
rs142426306
rs6751209
rs131797
rs61748181
rs78491606
rs150150565
rs73581419
rs1907702
rs11212631
rs1023767
rs185174247
rs56061761
rs6590343
rs41269079
rs10840270
rs4530278
rs2056726
rs111950327
rs9398196
rs7209057
rs2282764
All − MR Egger
All − Inverse variance weighted
−2
−1
0
1
2
MR effect size for
'telomere length || id:ieu−b−4879' on 'IDP_dMRI_TBSS_OD_Corticospinal_tract_L |
